# Upregulation of miR-376c-3p alleviates oxygen–glucose deprivation-induced cell injury by targeting ING5

**DOI:** 10.1186/s11658-019-0189-2

**Published:** 2019-12-04

**Authors:** Heng Zhang, Jie Zhou, Mingxia Zhang, Yanjie Yi, Bing He

**Affiliations:** Department of Pediatrics, Renmin Hospital of Wuhan University, Hubei Province, 430060 China

**Keywords:** miR-376c-3p, Oxygen–glucose deprivation, ING5, Cell cycle, Apoptosis

## Abstract

**Background:**

The expression level of miR-376c-3p is significantly lower in infants with neonatal hypoxic-ischemic encephalopathy (HIE) than in healthy infants. However, the biological function of this microRNA remains largely elusive.

**Methods:**

We used PC-12 and SH-SY5Y cells to establish an oxygen–glucose deprivation (OGD) cell injury model to mimic HIE in vitro. The miR-376c-3p expression levels were measured using quantitative reverse transcription PCR. The CCK-8 assay and flow cytometry were utilized to evaluate OGD-induced cell injury. The association between miR-376c-3p and inhibitor of growth 5 (ING5) was validated using the luciferase reporter assay. Western blotting was conducted to determine the protein expression of CDK4, cyclin D1, Bcl-2 and Bax.

**Results:**

MiR-376c-3p was significantly downregulated in the OGD-induced cell injury model. Its overexpression elevated cell viability and impaired cell cycle G0/G1 phase arrest and apoptosis in PC-12 and SH-SY5Y cells after OGD. Downregulation of miR-376c-3p gave the opposite results. We further demonstrated that ING5 was a negatively regulated target gene of miR-376c-3p. Importantly, ING5 knockdown had a similar effect to miR-376c-3p-mediated protective effects against cell injury induced by OGD. Its overexpression abolished these protective effects.

**Conclusion:**

Our data suggest that miR-376c-3p downregulated ING5 to exert protective effects against OGD-induced cell injury in PC-12 and SH-SY5Y cells. This might represent a novel therapeutic approach for neonatal HIE treatment.

## Background

Neonatal hypoxic-ischemic encephalopathy (HIE), which is also known as neonatal stroke, is caused by a disruption of cerebral blood vessels and leads to hypoxic or ischemic injury [1]. It is considered a major cause of disability in children after the neonatal period, with 0.1–0.2% incidence in term or near-term infants [2, 3]. Up to 40% of HIE patients usually suffer from devastating disability, including cerebral palsy, mental retardation, epilepsy and learning impairment [4–6]. Therapeutic hypothermia is the only recognized treatment for HIE, but it needs to be applied within 6 h of birth in a tertiary care center, which limits its application [7, 8]. Thus, it is important to better understand the molecular mechanisms of HIE.

MicroRNAs (miRNAs or miRs) are small endogenous non-coding RNAs that regulate a wide variety of biological processes, including differentiation, proliferation and apoptosis, by targeting mRNAs [9–11]. In recent years, researchers have found that miRs are closely associated with the pathogenesis of hypoxic-ischemic diseases. For example, miR-29b promotes neurocyte apoptosis by targeting MCL-1 during cerebral ischemia/reperfusion (I/R) [12]. MiR-451 has been reported to target CELF2, protecting against apoptosis and oxidative stress induced by oxygen and glucose deprivation/reoxygenation (OGD/R) [13]. Most recently, O′ Sullivan et al. found that the expression levels of three miRs (miR-374a-5p, miR-376c-3p and miR-181b-5p) are significantly lower in infants diagnosed with HIE than in healthy control infants [14]. This was determined by performing miRNA profile pattern analysis in umbilical cord whole blood.

Notably, miR-376c-3p has been shown to regulate cell growth, proliferation and migration in different cancer types [15, 16]. We thus speculated that miR-376c-3p might play an important role in neuronal cell survival under ischemic conditions.

The inhibitor of growth family member 5 (ING5) is composed of four molecular domains: a nuclear localization signal (NLS), a novel conserved region (NCR), a leucine zipper-like (LZL) domain, and a plant homeodomain (PHD) [17]. A related study indicated that ING5 is a key factor in DNA replication, cell cycle regulation and apoptosis [18]. ING5 overexpression could decrease cell proliferation and induce apoptosis in lung cancer [19] and esophageal squamous cell carcinoma [20]. Interestingly, Zhu et al. reported that ING5 suppresses cell viability and promotes cell apoptosis in human pulmonary artery smooth muscle cells under hypoxic conditions [21]. This highlights its potential for the treatment of hypoxic pulmonary hypertension. These results suggest that targeting ING5 might be beneficial for developing novel therapeutic strategies for HIE injury.

In this study, we constructed an OGD cellular model as the most commonly applied in vitro model of HIE [22, 23] to investigate the functional significance of miR-376c-3p in regulating neuron survival. Here, PC-12 [24–27] and SH-SY5Y [28] cells were used to construct an OGD cell injury model to mimic HIE. We confirmed whether miR-376c-3p exerted protective effects on OGD-injured cells. Furthermore, we explored the molecular mechanisms underlying miR-376c-3p in OGD cell injury.

### Materials and methods

#### Cell culture

PC-12 cells and SH-SY5Y cells were purchased from the American Type Culture Collection (ATCC) and cultured in Dulbecco’s modified Eagle medium (DMEM; HyClone) supplemented with 10% fetal bovine serum (FBS; Gibco). The culture was maintained at 37 °C in a humidified incubator containing 5% CO_2_.

### OGD cell injury model

Cells were cultured in glucose-free culture medium and placed into a hypoxia incubator with 94% N_2_, 5% CO_2_, 1% O_2_ for 2 h at 37 °C. Growth medium containing glucose was used to replace the culture medium and cells were cultured at 37 °C under normal conditions in an atmosphere with 5% CO_2_.

### Cell transfection

MiR-376c-3p mimic (mimic), miR-NC, anti-miR-376c-3p, anti-miR-NC, small interfering RNA targeting ING5 (si-ING5) and si-NC were all synthesized by GenePharma. The open reading frame of ING5 without its 3′-UTR was inserted into pcDNA3.1 vector (Sangon Biotech) to produce pcDNA3.1/ING5 vector. Lipofectamine 2000 (Invitrogen) was used to perform cell transfection prior to OGD.

### Quantitative reverse transcription PCR

Total RNA was isolated using Trizol Reagent and RNA was reverse transcribed using the TaqMan MicroRNA Reverse Transcription Kit or AMV reverse transcriptase random primers (Sigma-Aldrich) according to the manufacturer’s instructions. PCR was performed using TaqMan Universal Master Mix II (Sigma-Aldrich) for miR-376c-3p or SYBR Premix Ex Taq II kit (Takara) for ING5 with the following primer sequences: miR-376c-3p forward: 5′-AACATAGAGGAAATTCCACG-3′ and reverse: 5′-CAGTGCGTGTCGTGGAGT3′; U6 forward: 5′-CTCGCTTCGGCAGCACA-3′ and reverse: 5′-AACGCTTCACGAATTTGCGT-3′; ING5 forward: 5′-GGGAGATGATTGGCTGTG-3′ and reverse: 5′-CCTTTGGGTTTCGTGGTA-3′; GAPDH forward: 5′-AGAAGGCTGGGGCTCATTTG-3′ and reverse: 5′-CGATCCACACGGAGTACTTGC-3′.

The PCR amplification parameters were: 95 °C for 5 min, followed by 40 cycles of 95 °C for 15 s, 60 °C for 30 s and 72 °C for 30 s. The relative expression levels of miR-376c-3p and ING5 were calculated using the 2^−ΔΔCt^ method [29] with the respective internal controls U6 and GAPDH.

### Cell viability assay

Cells from different groups were seeded into 96-well plates (4 × 10^3^ cells per well) and incubated with 10 μl CCK-8 solution (Dojindo Laboratories) for 1 h. Using a Bio-Rad Microplate Reader, we measured the optical density values at 450 nm and used them to calculate relative cell viability in experimental groups compared with the control group.

### Flow cytometry analysis

Cells were collected and fixed at 4 °C with cold ethanol overnight. After two washes in phosphate-buffered saline (PBS), the cells were re-suspended in 200 μl binding buffer, followed by staining with 400 μl PI (BestBio) for 30 min in the dark. Next, the cell cycle distribution was analyzed via flow cytometry with FlowJo software (BD Bioscience).

To assess cell apoptosis, cells were collected, re-suspended and stained with Annexin V-FITC and PI (BestBio) for 20 min in the dark at room temperature. The numbers of early (Annexin V+/PI-), late (Annexin V+/PI+) and total apoptotic cells were determined using a flow cytometer equipped with CellQuest Pro software (BD Bioscience).

### Luciferase reporter assay

TargetScan Bioinformatics software (www.targetscan.org/vert_72) was searched to seek the putative target genes associated with the effects of miR-376c-3p on cell growth. For the luciferase reporter assay, the wild-type (WT) or mutant (MUT) 3′-untranslated region (3′-UTR) of ING5 was cloned into the pmirGLO dual luciferase reporter vectors (Promega) by RIBOBIO. These were transfected into HEK293T cells with mimic or miR-NC using Lipofectamine 2000 (Invitrogen). Cells were harvested after 48 h transfection and relative luciferase activities were determined using the Dual-Luciferase Reporter Assay System (Promega).

### Western blot analysis

RIPA lysis buffer and enhanced BCA Protein Assay kit (Beyotime) were respectively used to extract total protein and determine protein concentration. Approximately 30 μg of protein samples were separated using sulfate-polyacrylamide gel electrophoresis (SDS-PAGE) with 12% sodium dodecyl gel. The separated protein was transferred onto PVDF membranes where it underwent blocking with 5% nonfat milk for 2 h. Subsequently, the membranes were incubated with anti-ING5 and anti-GAPDH (Abcam) overnight at 4 °C, followed by incubation with horse radish peroxidase-labeled secondary antibody for 2 h at room temperature. The protein bands were visualized with GAPDH as an internal control using enhanced chemiluminescence (Pierce).

### Statistical analysis

Quantitative data were expressed as means ± SD from at least three experiments. GraphPad Prism 6.0 Software was used to perform statistical analysis. Differences were evaluated using Student’s t-test (2 groups) and one-way ANOVA followed by a Bonferroni post-hoc test (multiple groups). Values of p less than 0.05 were considered to be statistically significant.

### Results

#### The levels of miR-376c-3p decrease in the OGD-induced cell injury model

**Fig. 1:**
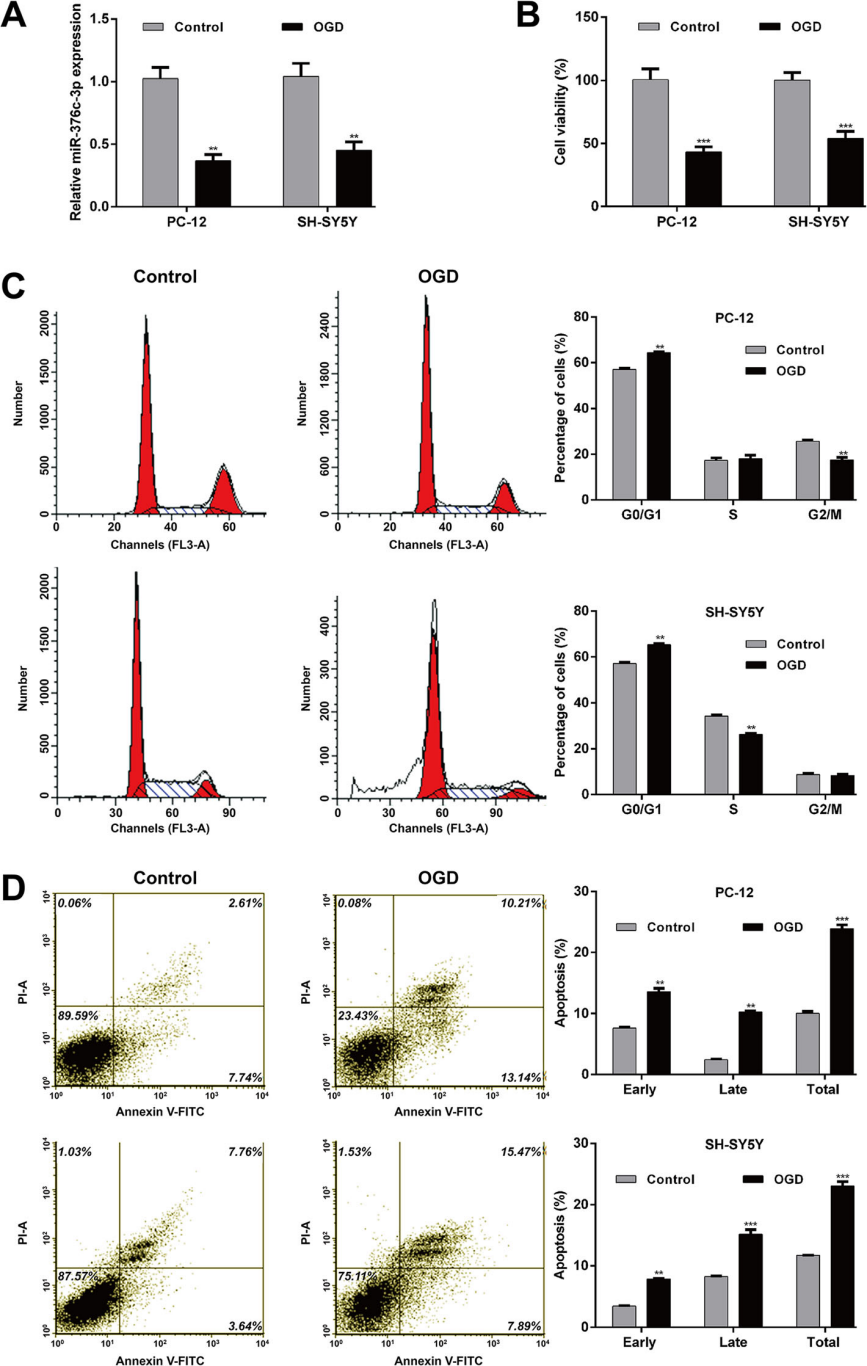
Expression of miR-376c-3p in the OGD-induced cell injury model. PC-12 and SH-SY5Y cells were subjected to OGD. Cells cultured under normal condition were used as the controls. (**a**) Quantitative reverse transcription PCR analysis of miR-376c-3p expression in PC-12 and SH-SY5Y cells. (**b**) Cell viability was measured using the CCK-8 assay. (**c**) Cell cycle distribution was analyzed via flow cytometry with PI staining. (**d**) Cell apoptosis was examined using flow cytometry with Annexin V/PI double staining. Data are expressed as means ± SD. **p < 0.01, ***p < 0.001 vs. control

PC-12 and SH-SY5Y cells in an OGD model were used to investigate the potential role of miR-376c-3p in HIE brain injury. MiR-376c-3p decreased significantly in PC-12 cells and SH-SY5Y cells after OGD (Fig. 1a, p < 0.01).

Then, we evaluated the OGD cell injury model. The CCK-8 assay showed that the cell viability of PC-12 and SH-SY5Y cells decreased significantly after OGD (Fig. 1b, p < 0.01). Moreover, the percentages of PC-12 cells and SH-SY5Y cells in G0/G1 phase increased significantly (*p* < 0.01), while the percentages of those in G2/M phase and S phase decreased after OGD (p < 0.01), indicating that OGD induced cell cycle G0/G1 phase arrest (Fig. 1c).

Furthermore, the percentage of apoptotic cells was remarkably elevated in the OGD group compared with the control group in both PC-12 and SH-SY5Y cells (Fig. 1d). These results reveal that downregulation of miR-376c-3p might play an important role in the OGD-induced cell injury model.

#### MiR-376c-3p significantly attenuates OGD-induced injury

**Fig. 2:**
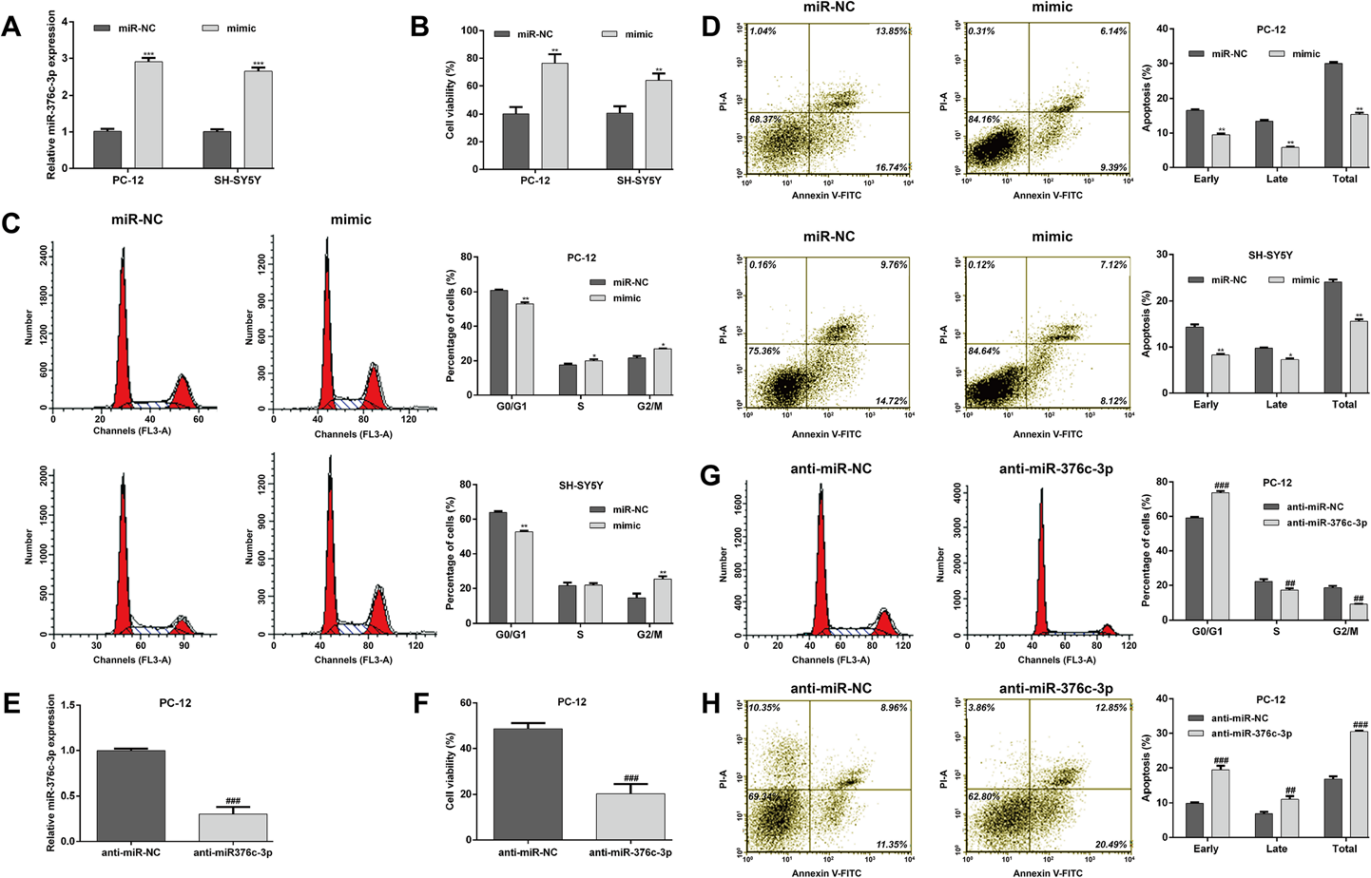
MiR-376c-3p attenuated OGD-induced cell injury. PC-12 and SH-SY5Y cells were transfected with miR-376c-3p mimic or miR-NC and then subjected to OGD. (**a**) Quantitative reverse transcription PCR analysis of miR-376c-3p expression in PC-12 and SH-SY5Y cells. (**b**) Cell viability was measured using the CCK-8 assay. (**c**) Cell cycle distribution was analyzed via flow cytometry with PI staining. (**d**) Cell apoptosis was examined using flow cytometry with Annexin V/PI double staining. (**e**) The expression of miR-376c-3p was analyzed in PC-12 cells after transfection with anti-miR-376c-3p or anti-miR-NC. (**f**) Cell viability was measured in PC-12 cells. (**g**) Cell cycle distribution was analyzed using flow cytometry with PI staining in PC-12 cells. (**h**) Cell apoptosis was examined using flow cytometry with Annexin V/PI double staining in PC-12 cells. Data are expressed as means ± SD. *p < 0.05, **p < 0.01, ***p < 0.001 vs. miR-NC; ^##^p < 0.01, ^###^p < 0.001 vs. anti-miR

We next performed gain-of-function assays in PC-12 and SH-SY5Y cells by transfection with mimic or miR-NC followed by OGD. Quantitative reverse transcription PCR showed that transfection with the mimic significantly upregulated the expression of miR-376c-3p in PC-12 and SH-SY5Y cells subjected to OGD (Fig. 2a, p < 0.001). The CCK-8 assay showed that miR-376c-3p overexpression significantly improved the viability of PC-12 and SH-SY5Y cells subjected to OGD (Fig. 2b, p < 0.01). Furthermore, OGD-induced cell cycle G0/G1 arrest (Fig. 2c) and apoptosis (Fig. 2d) was also significantly reversed upon miR-376c-3p overexpression.

We further confirmed the protective role of miR-376c-3p against OGD-induced injury with loss-of-function assays. Anti-miR-376c-3p transfection significantly suppressed the expression of miR-376c-3p in PC-12 cells (Fig. 2e). As expected, downregulation of miR-376c-3p promoted OGD-induced impaired cell viability (Fig. 2f), cell cycle G0/G1 phase arrest (Fig. 2g) and apoptosis (Fig. 2h) in PC-12 cells. These results suggest that miR-376c-3p exerts protective effects against OGD-induced injury.

#### ING5 is directly targeted by miR-376c-3p

**Fig. 3:**
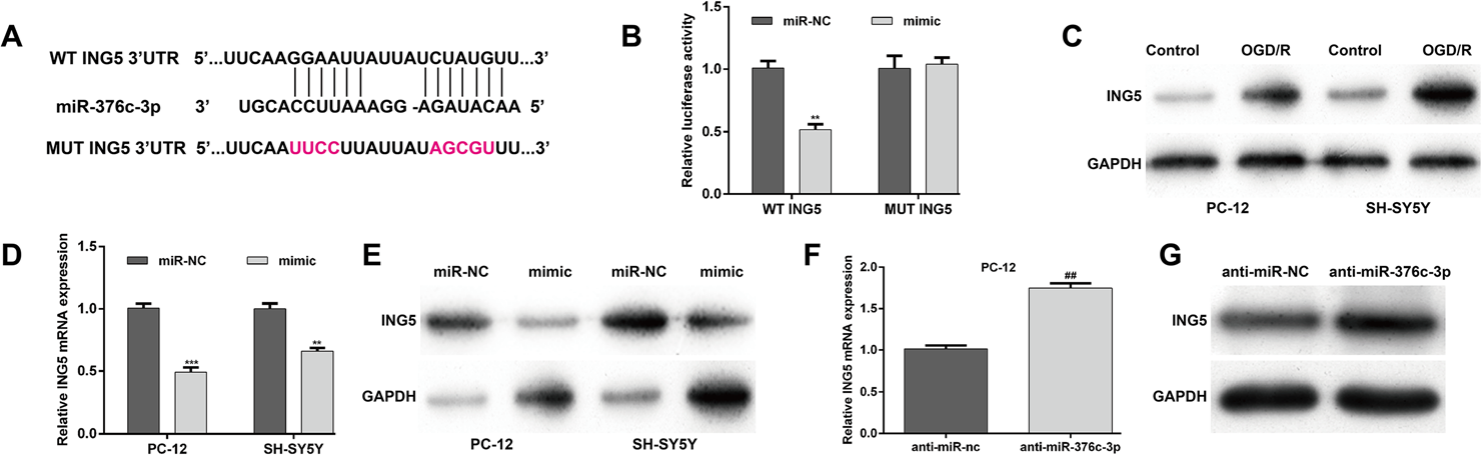
MiR-376c-3p targets the 3′-UTR of ING5. (**a**) Diagram of the predicted miR-376c-3p binding site in the 3′-UTR of ING5. (**b**) The luciferase reporter assay was performed to examine whether miR-376c-3p directly binds to the ING5 3′-UTR in HEK293T cells. (**c**) The protein expression of ING5 was analyzed in PC-12 and SH-SSY5Y cells subjected to OGD. **d** and **e**—The mRNA (**d**) and (**e**) protein expression levels of ING5 were measured in PC-12 and SH-SY5Y cells transfected with mimic or miR-NC and subjected to OGD. **f** and **g**—The mRNA (**f**) and (**g**) protein expression levels of ING5 were determined in PC-12 cells transfected with anti-miR-376c-3p or anti-miR-NC and subjected to OGD. Data are expressed as means ± SD. **p < 0.01 vs. miR-NC; ^##^p < 0.01 vs. anti-miR

Using bioinformatics analysis, we predicted the downstream target genes of miR-376c-3p and selected ING5, an important gene associated with cell growth, as a potential target gene of miR-376c-3p. The alignment of the seed regions of miR-376c-3p with the 3′-UTR of ING5 is shown in Fig. 3a.

The luciferase reporter assay was conducted to confirm direct target binding. Overexpression of miR-376c-3p significantly decreased the luciferase activity of a reporter vector containing the WT ING5 3′-UTR, but did not affect the luciferase activity of a reporter vector containing MUT ING5 3′-UTR in HEK293T cells (Fig. 3b, p < 0.01).

Subsequently, we analyzed the expression of ING5 in the OGD cell injury model using western blot analysis. The protein expression of ING5 was obviously elevated after OGD treatment in both PC-12 and SH-SY5Y cells (Fig. 3c). Furthermore, we demonstrated that overexpression of miR-376c-3p significantly decreased the mRNA (Fig. 3d) and protein (Fig. 3e) expression of ING5 in the OGD-induced PC-12 and SH-SY5Y cell injury model. By contrast, downregulation of miR-376c-3p elevated the mRNA (Fig. 3f) and protein (Fig. 3g) expression of ING5 in PC-12 cells. These results show that ING5 might be a direct target gene of miR-376c-3p.

#### Knockdown of ING5 and miR-376c-3p overexpression have similar protective effects against OGD-induced injury

**Fig. 4:**
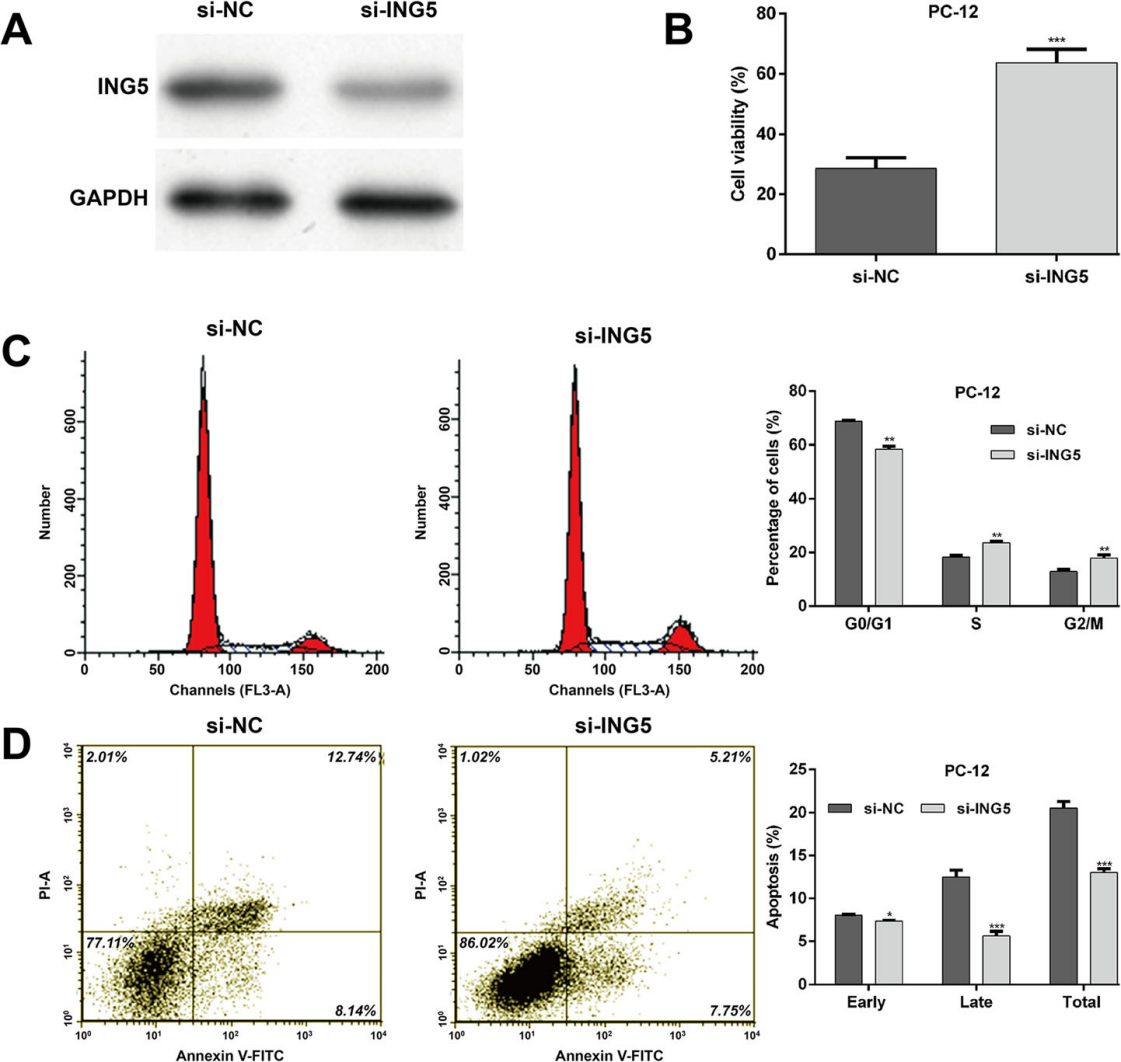
Knockdown of ING5 imitated the protective effect of miR-376c-3p against OGD-induced injury. PC-12 cells were transfected with si-ING5 or si-NC and then subjected to OGD. (**a**) The protein expression of ING5 was measured in PC-12 cells. (**b**) Cell viability was measured using the CCK-8 assay. (**c**) Cell cycle distribution was analyzed using flow cytometry with PI staining. (**d**) Cell apoptosis was examined using flow cytometry with Annexin V/PI double staining. *p < 0.05, **p < 0.01, ***p < 0.001 vs. si-NC

Since ING5 is negatively regulated by miR-376c-3p, we speculated that ING5 might promote OGD-induced injury. To validate our hypothesis, PC-12 cells were selected for transfection with si-ING5 for loss-of-function assays. The expression of ING5 protein was obviously downregulated in PC-12 cells after si-ING5 transfection (Fig. 4a). With an impact similar to miR-376c-3p overexpression, ING5 knockdown significantly reversed the impaired cell viability (Fig. 4b), cell cycle G0/G1 arrest (Fig. 4c) and apoptosis (Fig. 4d) induced by OGD treatment.

#### Restoration of ING5 expression reverses the protective effect of miR-376c-3p against OGD-induced injury

**Fig. 5:**
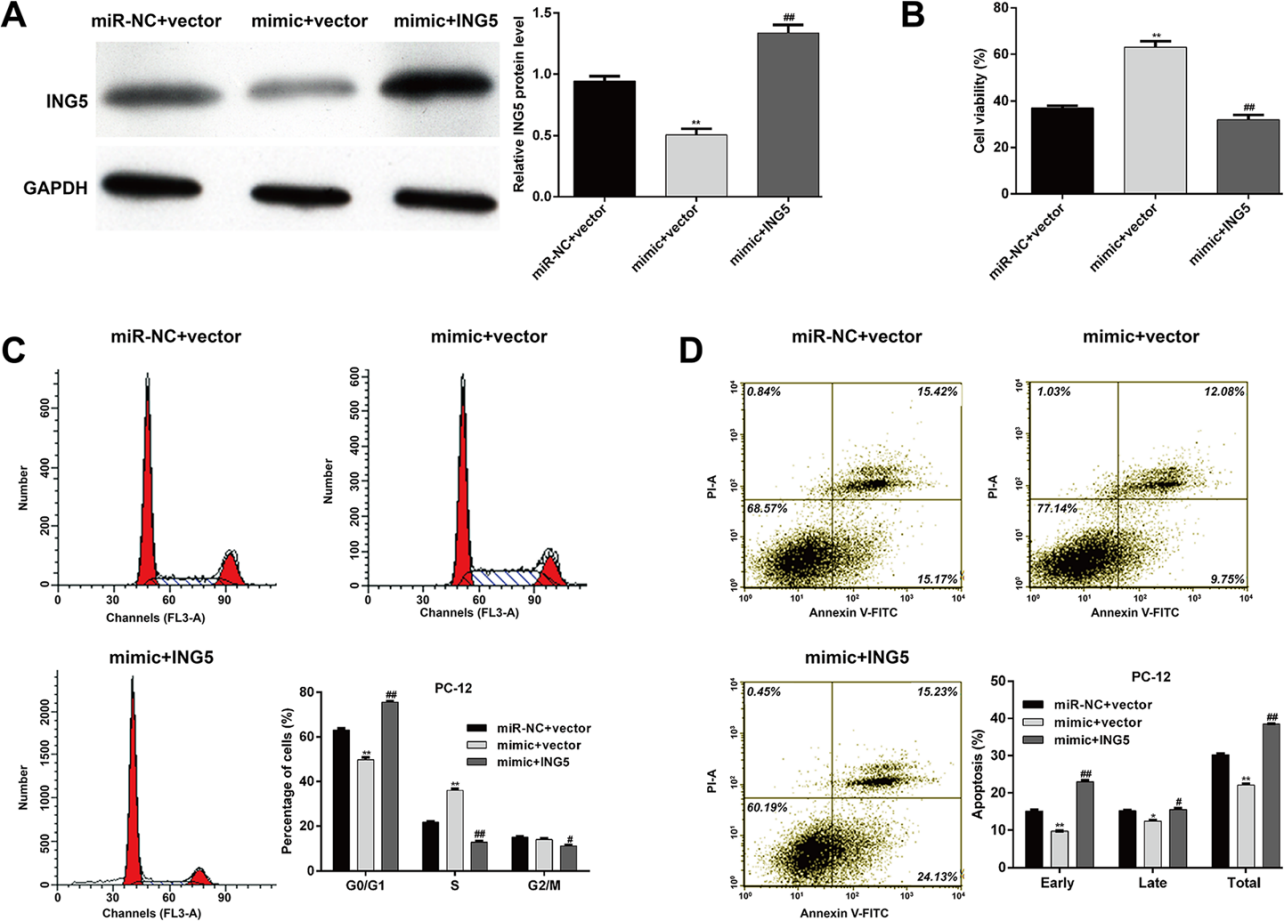
Restoration of ING5 expression reversed the effect of miR-376c-3p overexpression on OGD-induced cell injury. PC-12 cells were co-transfected with pcDNA3.1/ING5 vector and miR-376c-3p mimic and then subjected to OGD. (**a**) The protein expression of ING5 was detected using western blotting. (**b**) Cell viability was measured using the CCK-8 assay. (**c**) Cell cycle distribution was analyzed using flow cytometry with PI staining. (**d**) Cell apoptosis was examined using flow cytometry with Annexin V/PI double staining. Data are expressed as means ± SD. *p < 0.05, **p < 0.01 vs. miR-NC + vector; ^#^p < 0.05, ^##^p < 0.01 vs. mimic + vector

Next, we performed rescue experiments to confirm whether miR-376c-3p protects against OGD-induced cell injury by targeting ING5. ING5 expression was restored by transfection of ING5 plasmid into PC-12 cells that had undergone transfection with mimic. We first confirmed that the protein expression of ING5 was significantly restored by transfection with pcDNA3.1/ING5 vector (Fig. 5a, p < 0.01). The effect of miR-376c-3p overexpression on cell viability (Fig. 5b) was significantly blocked by restoration of ING5. In addition, the decrease in cell cycle G0/G1 phase arrest (Fig. 5c) and apoptosis (Fig. 5d) after miR-376c-3p overexpression were significantly abrogated by ING5 overexpression. These results suggest that ING5 might be a downstream functional regulator for miR-376c-3p-mediated protective effects in the OGD-induced cell injury model.

#### MiR-376c-3p regulates cell cycle arrest and apoptosis-associated factors by targeting ING5 in the OGD-induced cell injury model

**Fig. 6:**
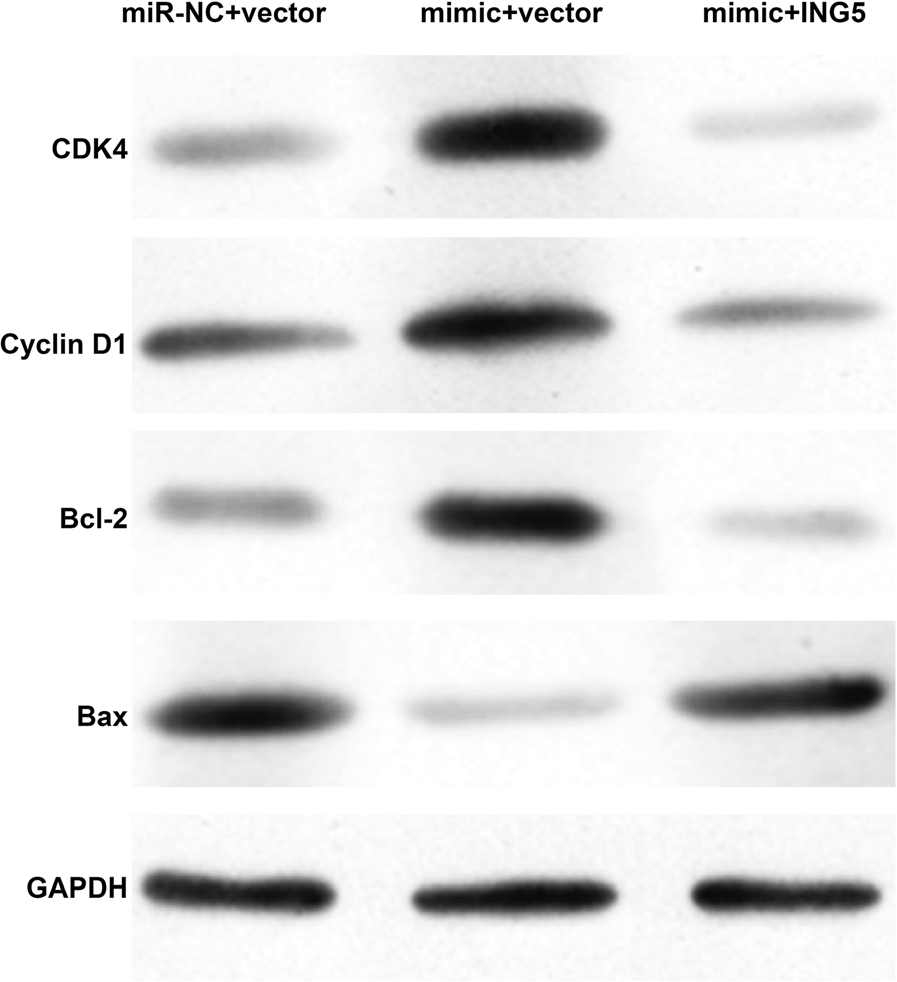
Restoration of ING5 expression alleviated the effect of miR-376c-3p overexpression on cell cycle arrest- and apoptosis-associated factors. PC-12 cells were co-transfected with pcDNA3.1/ING5 vector and miR-376c-3p mimic and then subjected to OGD. Western blot analysis was performed to measure the protein expressions of CDK4, cyclin D1, Bcl-2 and Bax. GAPDH was used as an internal control

Next, we analyzed the effects of miR-376c-3p and ING5 on the protein levels of cell cycle- and apoptosis-associated factors using western blot analysis. Compared with miR-NC + vector group, we found that miR-376c-3p overexpression significantly increased the protein levels of CDK4, cyclin D1 and Bcl-2, but decreased Bax expression in PC-12 cells subjected to OGD. Notably, the effects of miR-376c-3p overexpression on these protein levels were obviously alleviated by ING5 overexpression (Fig. 6). These findings further suggest that miR-376c-3p alleviates OGD-induced cell injury through downregulation of ING5.

## Discussion

MiR-376c-3p levels are significantly lower in infants diagnosed with HIE than in healthy control infants [14]. Consistently, we found that miR-376c-3p is significantly downregulated in response to OGD treatment. By performing gain-of-function and loss-of-function assays, we further found that miR-376c-3p significantly attenuates OGD-induced injury. The underlying mechanism for this might be reversal of cell cycle G0/G1 phase arrest and apoptosis, as confirmed by the upregulation of CDK4, cyclin D1 and Bcl-2 and downregulation of Bax after miR-376c-3p overexpression. Various studies have shown that miR-376c-3p is involved in regulating cell proliferation, cell cycle and apoptosis in neuroblastoma cells [16], gastric cancer [30] and hepatocellular carcinoma [15]. From this evidence, we hypothesized that miR-376c-3p might play a neuroprotective role in OGD-induced cell injury.

ING5 is the last member of the ING candidate tumor suppressor family that has been implicated in multiple cellular functions, including cell cycle regulation, apoptosis and chromatin remodeling [18]. Wu et al. [31] found that ING5 overexpression inhibits tumor growth in SH-SY5Y cells by suppressing proliferation and inducing apoptosis. In addition, ING5 has been reported as a potential target for breast cancer [32] and gastric cancer [33] treatment.

Our data show that the protein expression of ING5 is obviously elevated in OGD-induced cell injury. ING5 consistently and significantly aggravated hypoxic human pulmonary artery smooth muscle cells [21]. In fact, miRNA directly binds to the 3′-UTR of target mRNAs via complementary pairing sequences to induce their degradation [15].

We then explored whether ING5 is the downstream target gene of miR-376c-3p in OGD-induced cell injury. As expected, we found miR-376c-3p directly binds to the 3′-UTR of ING5. Moreover, ING5 knockdown imitated and ING5 overexpression reversed the protective effect of miR-376c-3p against OGD-induced injury. Furthermore, the regulatory effects of miR-376c-3p on CDK4, cyclin D1, Bcl-2 and Bax were abolished by ING5 overexpression. Similarly, ING5 is a target gene of miR-196a and suppresses head and neck cancer cell survival and proliferation [34]. Based on these data, we speculate that miR-376c-3p may downregulate ING5 expression in OGD-induced injury by regulating cell cycle and apoptosis-associated factors.

## Conclusions

Our experiments have confirmed our initial hypothesis that miR-376c-3p affects OGD-induced cell injury by targeting ING5. This study provides a theoretical basis for further investigation into the protection of neurons against OGD-induced injury. Of course, the impacts of other miRNAs on more target genes for HIE will be explored in future studies.

## Data Availability

The data in this study are available in this published article.

## Abbreviations

CCK-8: Cell Counting Kit-8
DMEM: Dulbecco’s modified Eagle medium
FBS: fetal bovine serum
HIE: Neonatal hypoxic-ischemic encephalopathy
HRP: horse radish peroxidase
ING5: inhibitor of growth family member 5
MUT: mutant
OGD: oxygen-glucose deprivation
SDS-PAGE: sodium dodecyl sulfate-polyacrylamide gel electrophoresis
WT: wild-type

## References

[1] Shellhaas RA, Kushwaha JS, Plegue MA, Selewski DT, Barks JD (2015). An evaluation of cerebral and systemic predictors of 18-month outcomes for neonates with hypoxic ischemic encephalopathy. J Child Neurol.

[2] Rao R, Trivedi S, Vesoulis Z, Liao SM, Smyser CD, Mathur AM (2017). Safety and short-term outcomes of therapeutic hypothermia in preterm neonates 34–35 weeks gestational age with hypoxic-ischemic encephalopathy. J Pediatr.

[3] Rong Z, Pan R, Chang L, Lee W (2015). Combination treatment with ethyl pyruvate and IGF-I exerts neuroprotective effects against brain injury in a rat model of neonatal hypoxic-ischemic encephalopathy. Int J Mol Med.

[4] du Plessis AJ, Volpe JJ (2002). Perinatal brain injury in the preterm and term newborn. Curr Opin Neurol.

[5] Pavithra L, Rutherford MA, Cowan FM (2009). Hypoxic-ischemic encephalopathy in preterm infants: antecedent factors, brain imaging, and outcome. Pediatr Res.

[6] Vannucci RC (2000). Hypoxic-ischemic encephalopathy. Am J Perinatol.

[7] Cerio FG, Lara-Celador I, Alvarez A, Hilario E (2013). Neuroprotective therapies after perinatal hypoxic-ischemic brain injury. Brain Sci.

[8] Chiang MC, Jong YJ, Lin CH (2017). Therapeutic hypothermia for neonates with hypoxic ischemic encephalopathy. Pediatr Neonatol.

[9] Flynt AS, Lai EC (2008). Biological principles of microRNA-mediated regulation: shared themes amid diversity. Nat Rev Genet.

[10] Xia HF, Jin XH, Cao ZF, Hu Y, Ma X (2014). MicroRNA expression and regulation in the uterus during embryo implantation in rat. FEBS J.

[11] Floris I, Kraft JD, Altosaar I (2016). Roles of microRNA across prenatal and postnatal periods. Int J Mol Sci.

[12] Huang Z, Lu L, Jiang T, Zhang S, Shen Y, Zheng Z, Zhao A, Gao R, Li R, Zhou S (2018). miR-29b affects neurocyte apoptosis by targeting MCL-1 during cerebral ischemia/reperfusion injury. Exp Ther Med.

[13] Liu Q, Hu Y, Zhang M, Yan Y, Yu H, Ge L (2018). microRNA-451 protects neurons against ischemia/reperfusion injury-induced cell death by targeting CELF2. Neuropsychiatr Dis Treat.

[14] O’Sullivan MP, Looney AM, Moloney GM, Finder M, Hallberg B, Clarke G, Boylan GB, Murray DM (2019). Validation of altered umbilical cord blood microRNA expression in neonatal hypoxic-ischemic encephalopathy. JAMA Neurol.

[15] Wang Y, Chang W, Chang W, Chang X, Zhai S, Pan G, Dang S (2018). MicroRNA-376c-3p facilitates human hepatocellular carcinoma progression via repressing AT-rich interaction domain 2. J Cancer.

[16] Bhavsar SP, Lokke C, Flaegstad T, Einvik C (2018). Hsa-miR-376c-3p targets Cyclin D1 and induces G1-cell cycle arrest in neuroblastoma cells. Oncol Lett.

[17] Yang XF, Shen DF, Zhao S, Ren TR, Gao Y, Shi S, Wu JC, Sun HZ, Zheng HC (2019). Expression pattern and level of ING5 protein in normal and cancer tissues. Oncol Lett.

[18] Campos EI, Chin MY, Kuo WH, Li G (2004). Biological functions of the ING family tumor suppressors. Cell Mol Life Sci.

[19] Liu XL, Meng J, Zhang XT, Liang XH, Zhang F, Zhao GR, Zhang T (2019). ING5 inhibits lung cancer invasion and epithelial-mesenchymal transition by inhibiting the WNT/beta-catenin pathway. Thorac Cancer.

[20] Zhang GJ, Zhao J, Jiang ML, Zhang LC (2018). ING5 inhibits cell proliferation and invasion in esophageal squamous cell carcinoma through regulation of the Akt/NF-kappaB/MMP-9 signaling pathway. Biochem Biophys Res Commun.

[21] Zhu TT, Sun RL, Yin YL, Quan JP, Song P, Xu J, Zhang MX, Li P (2019). Long noncoding RNA UCA1 promotes the proliferation of hypoxic human pulmonary artery smooth muscle cells. Pflugers Arch.

[22] Fernández-López D, Martínez-Orgado J, Casanova I, Bonet B, Leza JC, Lorenzo P, Moro MA, Lizasoain I (2005). Immature rat brain slices exposed to oxygen-glucose deprivation as an in vitro model of neonatal hypoxic-ischemic encephalopathy. J Neurosci Methods.

[23] Garnier Y, Middelanis J, Jensen A, Berger R (2002). Neuroprotective effects of magnesium on metabolic disturbances in fetal hippocampal slices after oxygen-glucose deprivation: mediation by nitric oxide system. J Soc Gynecol Investig.

[24] Cowan KM, Easton AS (2010). Neutrophils block permeability increases induced by oxygen glucose deprivation in a culture model of the human blood-brain barrier. Brain Res.

[25] Huang SL, He XJ, Li ZF, Lin L, Cheng B (2014). Neuroprotective effects of ginsenoside Rg1 on oxygen-glucose deprivation reperfusion in PC12 cells. Pharmazie.

[26] Li J, Qu Y, Chen D, Zhang L, Zhao F, Luo L, Pan L, Hua J, Mu D (2013). The neuroprotective role and mechanisms of TERT in neurons with oxygen-glucose deprivation. Neuroscience.

[27] Li W, Lou J, Wei L, Bai H, Zhang Y, He Y (2017). Ethyl pyruvate protects PC12 cells from oxygen-glucose deprivation: a potential role in ischemic cerebrovascular disease. Biomed Pharmacother.

[28] Liu Y, Eaton ED, Wills TE, McCann SK, Antonic A, Howells DW (2018). Human ischaemic cascade studies using SH-SY5Y cells: a systematic review and meta-analysis. Transl Stroke Res.

[29] Livak KJ, Schmittgen TD (2001). Analysis of relative gene expression data using real-time quantitative PCR and the 2 −ΔΔ C T method. Methods.

[30] Tu L, Zhao E, Zhao W, Zhang Z, Tang D, Zhang Y, Wang C, Zhuang C, Cao H (2016). hsa-miR-376c-3p regulates gastric tumor growth both in vitro and in vivo. BioMed Res Int.

[31] Wu JC, Jiang HM, Yang XH, Zheng HC (2018). ING5-mediated antineuroblastoma effects of suberoylanilide hydroxamic acid. Cancer Med.

[32] Zhao QY, Ju F, Wang ZH, Ma XZ, Zhao H (2015). ING5 inhibits epithelial-mesenchymal transition in breast cancer by suppressing PI3K/Akt pathway. Int J Clin Exp Med.

[33] Gou WF, Shen DF, Yang XF, Zhao S, Liu YP, Sun HZ, Su RJ, Luo JS, Zheng HC (2015). ING5 suppresses proliferation, apoptosis, migration and invasion, and induces autophagy and differentiation of gastric cancer cells: a good marker for carcinogenesis and subsequent progression. Oncotarget.

[34] Qin X, Guo H, Wang X, Zhu X, Yan M, Wang X, Xu Q, Shi J, Lu E, Chen W, Zhang J (2019). Exosomal miR-196a derived from cancer-associated fibroblasts confers cisplatin resistance in head and neck cancer through targeting CDKN1B and ING5. Genome Biol.

